# American Academy of Dental Science

**Published:** 1881-01

**Authors:** 


### *American Academy of Dental Science.*

The annual meeting of the American Academy of Dental Science was held October 27, in the lecture room of the Boston Society of Natural History at half past ten A. M. President J. L. Williams in the chair. The annual address was delivered by Dr. Joshua Tucker, of Boston ; it contained important results of more than forty years experience. Theories of decay and results from extracting teeth from crowded arches were among the important topics discussed. Dr. Brackett, of Newport followed with a paper on "The one thing needful to preserve filling." Discussion followed on the subjects of the address and essay by members of the Academy. It was voted that the thanks of the Academy be tendered Drs. Tucker and Brackett for their valuable essay. The annual dinner was served at Hotel Brunswick in the evening. The following offisers were elected for the ensuing year :—

J. L. WILLIAMS, *President*; J. H. CHANDLER, *Vice President*; J. T. CODMAN, *Recording Secretary*; C. P. WILSON, *Corresponding Secretary*; L. D. SHEPARD, *Treasurer*; H. F. BISHOP, *Librarian*; E. G. TUCKER, G. T. MOFFATT, F. N. SEABURY, *Censors.*

C. P. WILSON, *Cor. Sec'y.*

---

### EDITORIAL, ETC.

